# Accuracy of Canadian Food Labels for Sodium Content of Food

**DOI:** 10.3390/nu6083326

**Published:** 2014-08-22

**Authors:** Laura Fitzpatrick, JoAnne Arcand, Mary L’Abbe, Mengying Deng, Tara Duhaney, Norm Campbell

**Affiliations:** 1 Libin Cardiovascular Institute, University of Calgary, 3280 Hospital Drive NW, Calgary, AB T2N 4Z6, Canada; E-Mails: laura.fitzpatrick@uottawa.ca (L.F.); mden@ucalgary.ca (M.D.); tduhaney@ucalgary.ca (T.D.); 2 Department of Nutritional Science, Faculty of Medicine, University of Toronto, 150 College Street, Toronto, ON M5S 3E2, Canada; E-Mails: joanne.arcand@utoronto.ca (J.A.); mary.labbe@utoronto.ca (M.L.); 3 Medicine, Physiology and Pharmacology and Community Health Sciences, Libin Cardiovascular Institute, University of Calgary, 3280 Hospital Drive NW, Calgary, AB T2N 4Z6, Canada

**Keywords:** sodium, calories, saturated fatty acids, trans fatty acids, sugar, Canadian Food Inspection Agency (CFIA), Nutrition Facts table (NFt), food label accuracy, nutrition policy, monitoring population salt intake

## Abstract

The accuracy of the Nutrition Facts table (NFt) has a significant impact on Canadian efforts to reduce dietary sodium and monitor sodium content in foods. This study assessed the accuracy of sodium (and calories, trans fat, saturated fat, sugar) reported on the NFt for selected foods and beverages in Canada. The Canadian Food Inspection Agency (CFIA) sampled over 1000 foods and beverages from supermarkets, bakeries, and restaurants across Canada between January 2006 and December 2010. The samples were analyzed in CFIA laboratories. Results were requested for products with ≥1 of the following nutrients tested: sodium, calories, saturated fat, trans fat, and sugar. Differences between the label and laboratory values were calculated for each product. Overall, 16.7% (*n* = 169) of products were “unsatisfactory” with laboratory values exceeding ±20% of the NFt value. Sodium had the highest number of unsatisfactory products (*n* = 49, 18.4%) and trans fat had the lowest number of unsatisfactory products (*n* = 16, 4.3%). The proportion of unsatisfactory products for saturated fat, calories, and sugar was 15.8%, 14.2%, and 12.9%, respectively. All of the unsatisfactory products had excess nutrient content relative to the NFt. Sodium and calories were consistently underreported (*p* < 0.05), while NFt values for the other nutrients were not statistically different than laboratory values. Increased monitoring of NFt sodium values is recommended in order to increase consumer confidence in this nutrition tool, to encourage industry to accurately report nutrient content and to continue using the NFt to guide research, education, and policy development.

## 1. Introduction

Over 36 million deaths worldwide and 150,000 deaths in Canada can be attributed to non-communicable diseases (NCDs) including heart disease, stroke, diabetes, cancer and respiratory diseases [1]. NCD deaths are expected to increase by 15% globally between 2010 and 2020 [2]. Unhealthy diets and the consumption of foods high in salt, saturated fatty acids, trans fatty acids, and sugar are the cause of an estimated 40% of all NCD related deaths globally [3]. The increased availability of pre-packaged foods with unhealthy nutrient profiles is a driving force behind poor dietary habits worldwide [4]. The 2011 UN High-Level Meeting on NCDs highlighted the need for nutrition policies that promote the consumption of foods low in salt, saturated and trans fat, and sugar, to improve the lifespan and wellbeing of current and future generations [3]. In response to the UN recommendations, the WHO devised a set of nine voluntary global NCD targets for 2025, one that specifically focuses on reducing dietary salt/sodium intake by 30% [5]. In Canada, a major educational program has been launched to encourage Canadians to reduce dietary salt by reading the nutrition facts panel on pre-packaged foods [6].

The Nutrition Facts table (NFt) in Canada displays information on calories and 13 core nutrients (e.g., sodium, trans fat) and is present on virtually all pre-packaged foods and beverages. Amendments to the Food and Drugs Regulations in 2003 included mandatory NFt labels on most pre-packaged foods, providing consumers with the resources to make informed dietary decisions [7]. Food labels have consistently been cited as the number one source of nutritional information for Canadians, with 71% of consumers using the NFt [8]. Nutrition labels could also play an important role in the prevention and management of many chronic diseases, including diabetes and hypertension if they were accurate, available at point of purchase, and easy to understand [9]. Although two thirds of Canadians report using food labels, only 56% consider the information on food labels credible [10].

Canadians’ ability to make informed nutritional decisions is grounded in the assumption that the NFt is accurate and reliable. There are few studies assessing the accuracy of the NFt on food and beverages sold in Canada [10]; however, a 2011 media release suggested substantive inaccuracy of Canadian nutrition labels [11]. NFt inaccuracies could have serious implications for the many consumers who use this data to make healthy dietary choices and for decision makers developing and evaluating national nutrition policies. The primary goal of this investigation is to assess NFt accuracy for sodium, while also assessing the accuracy of other nutrients that have been identified as a major health concern (calories, saturated fat, trans fat, and sugar).

## 2. Experimental Section

### 2.1. Study Design

The Canadian Food Inspection Agency (CFIA) sampled and analyzed over 1000 foods and beverages from grocery stores, bakeries, and restaurants across Canada between 1 January 2006 and 31 December 2010. Products were selected according to regular surveillance and monitoring standards and to follow-up on consumer and trade complaints. The authors requested the CFIA data for those products in which one or more of the following nutrients was assessed: sodium, calories, saturated fat, trans fat, and/or sugar. It should be noted that most products were tested for 1–2 of the aforementioned nutrients. For example, some products were assessed for sodium only and some were tested for trans and saturated fat. The initial file (AL-2011-00049) was requested from the CFIA Access to Information division in April 2012. A second request for additional information (A-2012-0015) was submitted in August 2012 and the documentation was received in May 2013. The data from both requests was combined, for a total of 1014 products.

The information extracted from the CFIA files included product, food group classification and sub-classification, common name, brand, manufacturer, label claim, portion size, and NFt label and laboratory-measured values for each nutrient. Where there were discrepancies in food group classifications as defined by the CFIA (*i.e.*, “jam” was defined as a “fruit product” in some cases and as a “sweetener” in others), foods were re-classified according to the Schedule M groups found in the CFIA’s Guide to Food Labeling and Advertising [12]. All entries were reviewed to ensure the portion tested in the lab equaled the portion size on the label. For those products with discrepancies (*n* = 3), the laboratory measurement was manually corrected to account for the difference. There were no repeated entries noted during analysis, although some products were re-tested in subsequent years. Box plots were generated for each nutrient to determine the distribution and range of differences and to identify extreme data points. A total of four outliers (one for calories and three for sodium) were excluded following comparison with similar products in the Canadian Nutrient File.

### 2.2. CFIA Laboratory Analysis

The CFIA compliance test is used to assess the accuracy of nutrition labeling and claims. This testing involves analyzing the nutrient content of three composite samples consisting of four consumer units each, which are randomly selected from a single lot [13]. The mean nutrient content for the three composite samples is calculated and compared with the value reported on the NFt. The results are subjected to the principal CFIA acceptance criterion, requiring accuracy within 20% of the declared NFt value. A tolerance of 20% is permitted to account for variability in nutrient concentrations and to encourage manufacturers to label foods with the true average for the given lot [13]. Based on these criteria, products were assigned a specific conclusion as outlined in Table 1.

**Table 1:** The Canadian Food Inspection Agency (CFIA) compliance-based conclusions.

Conclusion	CFIA Definition
Unsatisfactory	Lab results exceeded label value by >20%
Investigative	Non-compliant with CFIA rounding rules
Satisfactory	Lab results were accurate within 20% of the label value
No Decision	No NFT available

### 2.3. Statistical Analysis

The frequency of each CFIA compliance-based conclusion was calculated for the overall data set and for each nutrient using SAS/STAT^®^ 9.3 software. The difference between the label and laboratory-measured value was calculated for each product. This difference could not be calculated for some products (*n* = 13), as the label value was not included in the Food Submission Document. These products were only included in the analysis of CFIA conclusions (*i.e.*, “unsatisfactory”, “satisfactory”, *etc.*) and were not part of the mean calculations. The mean difference, standard deviation, and confidence limits were computed for each nutrient category. A sub-analysis was performed to determine the mean difference for: (1) products that overstate nutrient content (*i.e.*, negative differences) and (2) products that understate nutrient content (*i.e.*, positive differences). Paired *t*-tests were conducted to determine statistical significance (*p* < 0.05) between the mean label and mean laboratory values at the 95% confidence level. Categorical data are presented as frequencies and percentages, while continuous data is presented as means and 95% confidence intervals.

## 3. Results

### 3.1. Analysis of CFIA Conclusions by Nutrient

After omitting outliers, a total of 1010 products were assessed. Overall, 169 products (16.7%) products were “unsatisfactory” for at least one nutrient, while 777 products (76.9%) were “satisfactory” for all nutrients tested (Table 2). The remaining products were classified as “investigative” (4.4%) and “no decision” (2.0%), indicating that further testing is required to confirm CFIA laboratory results.

**Table 2:** Frequency of CFIA conclusions overall and for each nutrient.

Nutrients	Unsatisfactory		Satisfactory		Investigative		No Decision		Total	
	*n*	%	*n*	%	*n*	%	*n*	%	*n*	%
Overall	169	16.7	777	76.9	44	4.4	20	2.0	1010	100.0
Sodium	49	18.4	200	75.2	15	5.6	2	0.8	266	100.0
Calories	31	14.2	169	77.2	11	5.0	8	3.7	219	100.0
Saturated Fat	60	15.8	298	78.4	17	4.5	5	1.3	380	100.0
Trans fat	16	4.3	347	92.3	3	0.8	10	2.7	376	100.0
Sugar	17	12.9	112	84.9	2	1.5	1	0.8	132	100.0

Table 2 outlines CFIA conclusions with respect to nutrient category. Sodium had the highest number of unsatisfactory products (*n* = 49, 18.4%) and trans fat had the lowest number of unsatisfactory products (*n* = 16, 4.3%). The proportion of unsatisfactory products for saturated fat, calories, and sugar was 15.8%, 14.2%, and 12.9%, respectively. All of the “unsatisfactory” products contained excess nutrients over that indicated in the NFt.

### 3.2. Comparing Label and Laboratory Values: Mean Differences

Table 3 depicts the mean differences and associated confidence intervals for each nutrient. The mean difference for sodium products (*n* = 266) was 17.6 [5.9,29.2] mg/portion (*p* < 0.0032). The mean difference for calories (*n* = 219) was 10.6 [7.0,14.1] kcal/portion (*p* < 0.0001). The mean difference for saturated (*n* = 380) and trans (*n* = 376) fat was 0.07 [−0.01,0.15] g/portion and 0.02 [−0.03,0.06] g/portion, respectively. For sugar (*n* = 132), the mean difference was 0.1 [−0.3,0.5] g/portion. The mean difference was not statistically significant for saturated fat, trans fat, and sugar.

**Table 3:** Comparing label and laboratory values: Means and 95% confidence intervals.

Nutrients	Label Value		Laboratory Value		Difference		*p*-Value
	Mean	95% CI	Mean	95% CI	Mean	95% CI	
*Sodium (mg/portion)*							
Overall (*n* = 266)	268.4	(229.7,307.1)	285.9	(245.8,326.0)	17.6	(5.9,29.2)	0.003
Positive (*n* = 142)	277.0	(225.4,328.5)	339.2	(281.7,396.6)	62.2	(44.4,80.0)	
Negative (*n* = 122)	261.9	(202.0,321.7)	227.8	(172.9,282.7)	−34.1	(−41.7,−26.4)	
*Calories*							
Overall (*n* = 219)	132.2	(120.1,144.3)	142.7	(130.4,155.1)	10.6	(7.0,14.1)	<0.001
Positive (*n* = 152)	130.1	(116.2,144.0)	150.2	(135.4,165.1)	20.1	(16.1,24.1)	
Negative (*n* = 62)	137.6	(112.1,163.0)	125.5	(102.5,148.5)	−12.0	(−16.3,−7.8)	
*Saturated Fat (g/portion)*							
Overall (*n* = 380)	1.47	(1.26,1.67)	1.54	(1.34,1.73)	0.07	(−0.01,0.15)	0.095
Positive (*n* = 212)	1.08	(0.84,1.31)	1.47	(1.20,1.74)	0.40	(0.29,0.50)	
Negative (*n* = 162)	2.02	(1.66,2.38)	1.66	(1.36,1.96)	−0.36	(−0.45,−0.27)	
*Trans fat (g/portion)*							
Overall (*n* = 376)	0.15	(0.09,0.22)	0.17	(0.10,0.24)	0.02	(−0.03,0.06)	0.460
Positive (*n* = 291)	0.06	(0.01,0.11)	0.15	(0.07,0.23)	0.09	(0.06,0.12)	
Negative (*n* = 41)	0.95	(0.55,1.36)	0.47	(0.22,0.73)	−0.48	(−0.76,−0.20)	
*Sugar (g/portion)*							
Overall (*n* = 132)	7.0	(5.4,8.7)	7.1	(5.5,8.7)	0.1	(−0.3,0.5)	0.599
Positive (*n* = 64)	5.6	(3.7,7.5)	7.0	(4.9,9.1)	1.4	(0.8,2.0)	
Negative (*n* = 54)	10.5	(7.3,13.6)	9.1	(6.2,11.9)	−1.4	(−1.9,−0.9)	

### 3.3. Graphical Analysis: Distribution of Differences for Sodium and Calories

**Figure 1:**
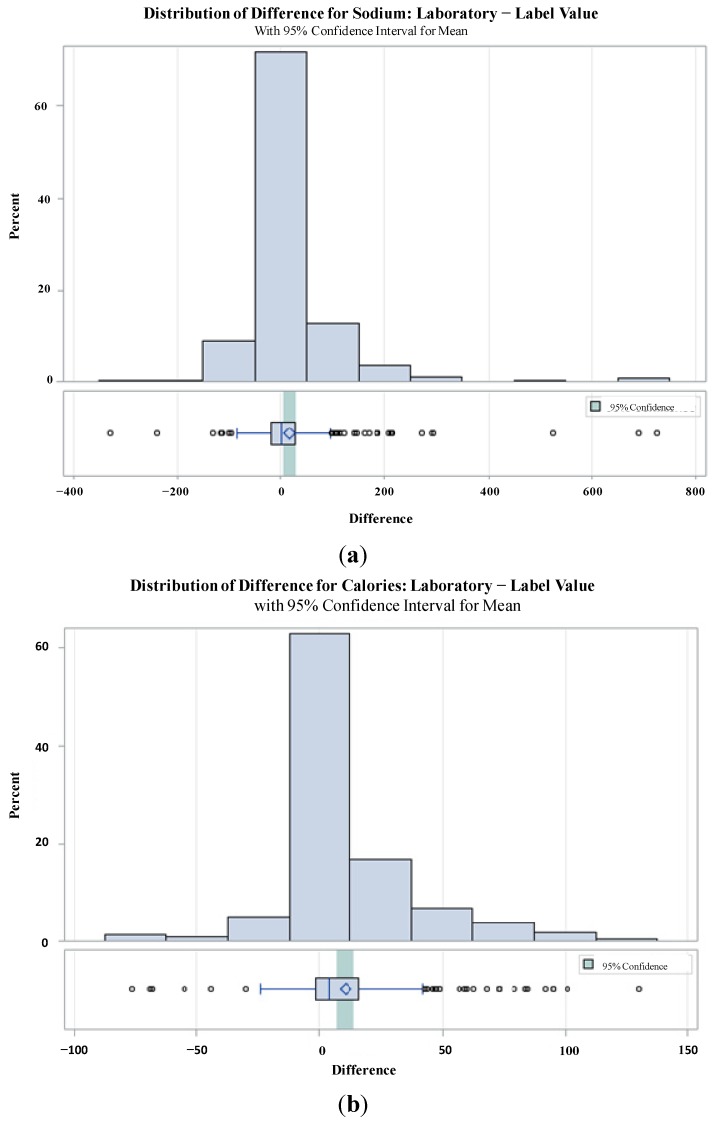
Distribution of differences (laboratory value − label value) for (**a**) sodium and (**b**) calories.

The mean, 95% confidence interval for the mean, and extreme data points fare illustrated in Figure 1 for sodium (a) and calories (b). The most extreme differences were observed for sodium, with nine products exceeding the label value by greater than 200 mg/portion. For calories, there were 17 labels that understated the nutrient content by more than 50 kcal/portion, compared to five labels that overstated the calorie content by the same amount.

* The data are presented as frequency distributions in bar graphs and box plots. In the box plots, the median is represented by a vertical line, the mean is denoted by a diamond shape, and the first and third quartile are the represented by the sides of the box. The extreme differences are represented by individual data points extending beyond the whiskers (1.5 IQR).

### 3.4. Frequency of “Unsatisfactory” Products by Food Category

For each food category, the number of products tested was tabulated, including the frequency of “unsatisfactory” products (Table 4). The majority of products sampled in this investigation were “bakery products” (*n* = 363), “snacks” (*n* = 102), and “sugar & sweets” (*n* = 95). The proportion of unsatisfactory bakery products was 19.6%, which was considerably higher than the number of unsatisfactory products in snacks (11.8%) and sugar/sweets (9.5%). Other categories with a large proportion of unsatisfactory products include “dairy products & substitutes” (*n* = 58, 20.7%) and “sauces, gravies, dips, and condiments” (*n* = 45, 20.0%). There were several food categories where greater than 30% of products tested were unsatisfactory; however, the sample size for these food categories was small (e.g., “potatoes & yams”, *n* = 6, 66.7% unsatisfactory).

**Table 4:** Frequency of unsatisfactory products by food category.

Food Category	Total		Unsatisfactory	
	*N*	%	*N*	%
Bakery products	363	35.9	71	19.6
Beverages	21	2.1	2	9.5
Cereals & other grain products	70	6.9	8	11.4
Dairy products & substitutes	58	5.7	12	20.7
Desserts	16	1.6	3	18.8
Dessert toppings & fillings	0	0.0	0	0.0
Egg & egg substitutes	0	0.0	0	0.0
Fats & oils	48	4.8	3	6.3
Marine & fresh water animals	12	1.2	2	16.7
Fruit & fruit juices	26	2.6	2	7.7
Legumes	9	0.9	2	22.2
Meat, poultry, products, substitutes	11	1.1	3	27.3
Miscellaneous	8	0.8	3	37.5
Combination dishes	21	2.1	7	33.3
Nuts & seeds	20	2.0	3	15.0
Potatoes, sweet potatoes, & yams	6	0.6	4	66.7
Salads	0	0.0	0	0.0
Sauces, dips, gravies, condiments	45	4.5	9	20.0
Snacks	102	10.1	12	11.8
Soups	64	6.3	9	14.1
Sugar & sweets	95	9.4	9	9.5
Vegetables	15	1.5	5	33.3
Total	1010	100.0	169	16.7

## 4. Discussion

This investigation suggests that the NFt is accurate for the majority of products available at Canadian grocers, bakeries, and restaurants. Of the 1010 products analyzed, 16.7% (*n* = 169) were “unsatisfactory”, with laboratory results greater than 20% of the declared value on the NFt. Laboratory analysis showed that the actual sodium and calorie content of foods were 17.6% and 10.6% higher, respectively, as compared to the levels reported on the NFt; therefore, there was a tendency for the NFt to underestimate sodium and calorie content in the foods and beverages tested in this investigation. In fact all of the products defined as unsatisfactory had excess nutrient content relative to the NFt. Of the food categories with a larger sample size, “bakery products”, “dairy products and substitutes”, and “sauces/dips/gravies” had nearly one in five products testing unsatisfactory. The inaccuracy of bakery food labels may be due to difficulties standardizing ingredients and preparation on a day-to-day basis. In addition, smaller, private food manufacturers may not have access to advanced laboratories or funding to support in-depth nutrient analysis. However, these technical challenges should not have resulted in such large discrepancies between the label and laboratory values.

The primary purpose of the NFt is to communicate essential nutritional information to Canadians, enabling consumers to make healthy food choices. Approximately three in four Canadians view nutrition labels as an important source of information and nearly 80% of consumers have used the NFt to compare food products [14]. However, probably as a result of media on the inaccuracy of the food label, only 56% of consumers consider food labels to be an accurate and credible source of information [10]. Inaccurate NFTs could have serious implications for consumers who rely on food labels to inform their dietary decisions. For example, based on the results of this investigation, Canadians who consume a diet high in pre-packaged foods may be underestimating their daily sodium intake. This is especially true for individuals on low sodium diets for the management of chronic diseases [15]. NFt data also forms the basis of consumer tools to support healthy eating. Cell phone applications that analyze dietary intake using NFt data may inaccurately estimate daily consumption patterns.

Several national nutrition policies have been developed and evaluated using NFt information. Health Canada also used NFt data to provide benchmarks for the food industry in an effort to lower the sodium levels of processed foods sold in Canada [16]. The International Food Monitoring Group, run out of the George Institute in Australia, is coordinating a global nutrient database focused on dietary sodium that monitors and compares the nutritional composition of foods across countries [17]. The Nutritional Science Department at the University of Toronto is also presently using NFt label data to conduct research focused on sodium in the food supply, which will be used to evaluate and inform nutrition policies [18]. The Trans Fat Monitoring Program introduced in June 2007 also used a combination of laboratory and NFt data [19].

There is minimal literature assessing the accuracy of the NFt in Canada. A 2011 study by Pantazopoulus *et al.* investigated the accuracy of trans and saturated fat values reported on the NFt for specific foods (cookies, crackers, granola bars, breakfast bars, frozen foods) collected between 2005 and 2008 as part of Canada’s Trans Fat Monitoring Program [10]. The authors reported no significant difference between label and laboratory values for trans fat (mean difference = −0.08 ± 0.50) and saturated fat (mean difference = 0.13 ± 0.75). These findings are in agreement with the current results, where there was no substantive mean difference for trans and saturated fat. The success of trans fat reporting on nutrition labels could be attributed to the introduction of the Health Canada Trans Fat Monitoring Program and closer industry attention to label accuracy [19].

There are several limitations of this investigation that must be addressed. Although the data included a wide range of food and beverages, with the majority selected according to regular CFIA surveillance procedures, the results may not be generalizable to all products in the Canadian marketplace. In addition, each sample was only representative of a single lot tested at a single point in time. The outliers and extreme data may be attributed to lot variability, such as seasonal ingredient changes or differences in laboratory analytic techniques. Analysis and comparison of NFt accuracy with respect to food category was also problematic due to variable sample sizes (e.g., over 300 “bakery products” tested *vs.* nine products tested in “legumes”). More thorough and systematic testing of products should be conducted, especially in those food categories with small sample sizes and large inaccuracies. The data was collected and distributed by the CFIA, and there was minimal access to CFIA resources and documentation and limited ability to communication with the individuals conducting the sample testing and analysis. More recently, the CFIA rather than enhancing monitoring has ceased monitoring of the accuracy of the food label.

## 5. Conclusions

This investigation provides a snapshot of food labeling accuracy for sodium and other nutrients on the NFt according to testing conducted by the CFIA. The results suggest that the NFt is accurate for the majority of food and beverages available to Canadian consumers; however, enhanced monitoring and regulation of sodium values reported in the NFt are necessary to support existing sodium reduction strategies and to encourage the food industry to accurately label. Improving policies and legislation that target food labeling practices and allocating adequate resources for monitoring will help support Canadians’ efforts to make healthier food choices and reduce their risk of diet-related NCDs.
